# Baseline Characteristics in the Remote Diet Intervention to REduce long-COVID Symptoms Trial (ReDIRECT)

**DOI:** 10.3310/nihropenres.13522.1

**Published:** 2024-03-05

**Authors:** Laura Haag, Janice Richardson, Caroline Haig, Yvonne Cunningham, Heather Fraser, Naomi Brosnahan, Tracy Ibbotson, Jane Ormerod, Chris White, Emma McIntosh, Kate O'Donnell, Naveed Sattar, Alex McConnachie, Mike Lean, David Blane, Emilie Combet

**Affiliations:** 1 Human Nutrition, School of Medicine, Dentistry & Nursing, University of Glasgow, Glasgow, Scotland, G31 2ER, UK; 2 School of Cardiovascular and Metabolic Health, University of Glasgow, Glasgow, Scotland, G12 8TA, UK; 3 Robertson Centre for Biostatistics, School of Health and Wellbeing, University of Glasgow, Glasgow, Scotland, G12 8TA, UK; 4 General Practice & Primary Care, School of Health and Wellbeing, University of Glasgow, Glasgow, Scotland, G12 8TA, UK; 5 Health Economics and Health Technology Assessment, School of Health and Wellbeing, University of Glasgow, Glasgow, Scotland, G12 8TA, UK; 6 Counterweight Ltd., Abergavenny, UK; 7 Long Covid Scotland, UK, UK

**Keywords:** Long COVID, Weight management, Diet, Remote delivery, Personalisation, Post COVID-19 Syndrome, Fatigue, Pain, Breathlessness

## Abstract

**Background:**

The persistence of symptoms for ≥12 weeks after a COVID-19 infection is known as Long COVID (LC), a condition with unclear pathophysiology and no proven treatments to date. Living with obesity is a risk factor for LC and has symptoms which may overlap with and aggravate LC.

**Methods:**

ReDIRECT is a remotely delivered trial assessing whether weight management can reduce LC symptoms. We recruited people with LC and BMI >27kg/m ^2^. The intervention was delivered remotely by dietitians, with online data collection (medical and dietary history, COVID-19 infection and vaccination, body composition, LC history/symptoms, blood pressure, quality of life, sociodemographic data). Participants self-selected the dominant LC symptoms they most wanted to improve from the intervention.

**Results:**

Participants (n=234) in England (64%) and Scotland (30%) were mainly women (85%) of white ethnicity (90%), with 13% living in the 20% most deprived areas, a mean age of 46 (SD10) years, and median BMI of 35kg/m ^2^ (IQR 32-40). Before starting the study, 30% reported more than one COVID-19 infection (82% confirmed with one or more positive tests). LC Diagnosis was mainly by GPs (71%), other healthcare professionals (9%), or self-diagnosed (21%). The median total number of symptoms was 6 (IQR 4–8). Self-selected dominant LC symptoms included fatigue (54%), breathlessness (16%), pain (12%), anxiety/depression (1%) and "other" (17%). At baseline, 82% were taking medication, 57% reported 1+ other medical conditions. Quality of life was poor; 20% were on long-term sick leave or reduced working hours. Most (92%) reported having gained weight since contracting COVID-19 (median weight change +11.5 kg, range -11.5 to +45.3 kg).

**Conclusions:**

Symptoms linked to LC and overweight are diverse and complex. Remote trial delivery enabled rapid recruitment across the UK yet certain groups (e.g. men and those from ethnic minority groups) were under-represented.

**Trial registration:**

ISRCTN registry ( ISRCTN12595520, 25/11/2021).

## Plain Language summary

Long COVID (LC, symptoms lasting 12 weeks or more after a COVID-19 infection) is a poorly understood condition, with no proven treatments. Living with obesity increases the risk of developing LC; symptoms of obesity overlap and aggravate those of LC.

The ReDIRECT study tests, in people living with both LC and overweight, whether weight management can reduce LC symptoms. The study involves total diet replacement (with porridge, soups and shakes) for 12 weeks and is delivered remotely, with dietitian support via internet and/or phone. Researchers collected all data via online forms (medical and diet history, COVID-19 infection and vaccination, weight, height, LC history and symptoms, blood pressure, quality of life, and other demographic data). Each participant selected the LC symptom they most wanted to see improve.

Participants (n=234) lived across the UK, were mainly women (85%) of white ethnicity (90%), with 13% living in the 20% most deprived areas. Their average age was 46 years old with an average body mass index (BMI) of 35kg/m ^2^.

Diagnosis of LC was mainly by GPs (71%), other healthcare professionals (9%), or self-diagnosed (21%). Participants reported on average 6 symptoms each, identifying fatigue (54%), breathlessness (16%), pain (12%), anxiety/depression (1%) and "other" (17%) as the symptom they would most like to see improve.

At the start of the study, most (82%) were taking medication, half (57%) reported 1+ other medical conditions. Quality of life was poor, and 20% were on long-term sick leave or reduced working hours. Most (92%) reported gaining weight since contracting COVID-19, on average +11.5 kg.

The baseline characteristics of ReDIRECT study participants show that symptoms linked to LC and overweight are diverse and complex. The study being “remote” means that recruitment was rapid and across the UK, yet certain groups (e.g. men and those from ethnic minority groups) were under-represented.

## Introduction

The term' Long COVID' (LC) refers to prolonged symptoms following infection with SARS-CoV-2, the viral cause of COVID-19, that are not explained by an alternative diagnosis ( Greenhalgh *et al.*, 2022). Other names include 'post-COVID-19 syndrome' ( National Institute for Health and Care Excellence, 2021), 'post COVID-19 condition' ( Soriano *et al.*, 2022), 'post-covid conditions' ( Centers for Disease Control and Prevention, 2023), and post-acute sequelae of SARS CoV-2 infection (PASC) ( Horberg *et al.*, 2022). LC is a multi-system condition that affects approximately 10% of people following COVID-19 infection. However, estimates vary and may be lower following vaccination and with more recent COVID-19 variants ( Ballering *et al.*, 2022) ( Office for National Statistics, 2022).

LC is characterised by a constellation of symptoms, with some studies phenotyping the condition based on symptom clusters ( Evans *et al.*, 2022). For people living with LC, symptoms can vary in duration, severity, and impact on daily functioning. LC can follow an unpredictable, relapsing and remitting course, with 'flare-ups' following particular triggers (e.g. physical exertion) ( Brown & O'Brien, 2021; Hastie *et al.*, 2023). Among the most common symptoms are fatigue, breathlessness, pains, cognitive dysfunction, loss of taste/smell, and anxiety/depression ( Davis *et al.*, 2021; Sudre *et al.*, 2021), though over 50 symptoms have been identified ( Lopez-Leon *et al.*, 2021). The long-term impact of LC symptoms on the wellbeing and quality of life of people affected by this condition is unknown, nor is the economic impact.

The underlying pathophysiology of LC is not well understood, with several – likely interacting – proposed mechanisms. These include viral factors (e.g., viral persistence, reactivation); host factors (e.g., chronic inflammation, metabolic and endocrine dysregulation, autoimmunity); and downstream impacts (e.g., tissue damage from the initial infection, tissue hypoxia, and autonomic nervous system dysfunction) ( Castanares-Zapatero *et al.*, 2022; Davis *et al.*, 2023).

Comorbidities, such as cardiovascular disease, diabetes, obesity, and respiratory conditions, have been identified as risk factors for COVID-19. However, their influence on LC symptoms is not well understood. Moreover, there are also substantially higher odds of LC based on sex (female), higher deprivation and occupation (especially education or health-related occupations) ( Shabnam *et al.*, 2023).

Lifestyle factors, such as physical activity levels and dietary patterns, may influence LC symptoms, yet their association remains understudied. At the time of writing (02/11/2023), 501 trials investigating LC treatments were registered on clinicaltrials.gov and ISRCTN since 2020 (search terms included Long COVID and synonyms named above). Around 2/3 of the studies were interventional, focusing mainly on rehabilitation and alleviating individual symptoms. With only 26 studies investigating dietary approaches, mostly dietary supplements, dietary strategies to manage LC lack a solid evidence base. Results are awaited for most of these trials.

Weight increase was widely reported across populations following the first COVID-19 lockdown period (March–May 2020) ( Bakaloudi *et al.*, 2022), compounding an existing high prevalence of overweight and obesity in the general population – in the UK, 64% of all adults were reported to have a BMI greater than 25kg/m ^2^ in 2021 ( Organisation for Economic Co-operation and Development, 2021). Generally, body composition and weight changes post-COVID have not been thoroughly described in people living with LC. However, the EPILOC study highlighted a consistent association between increasing BMI and post-COVID fatigue, neurocognitive impairment, and chest symptoms, with the greatest recovery rate in those with BMI between 21 and 22 kg/m ^2^ ( Peter *et al.*, 2023).

Weight management programmes in adults with overweight/obesity have been reported to reduce symptoms such as fatigue, breathlessness, and pain, which are also common with LC (pain and QoL ( Höchsmann *et al.*, 2022), dyspnoea ( Bernhardt & Babb, 2014; Riddle & Stratford, 2013; Stenius-Aarniala *et al.*, 2000). However, the effectiveness of intentional weight loss to reduce symptoms of LC and prevent future cardiometabolic ill-health has not been studied despite that group's specific needs.

The Remote Diet Intervention to Reduce Long-COVID Symptoms Trial (ReDIRECT) tests the impact of an evidence-based dietary weight management programme on LC symptoms in people with overweight or obesity. Here, we describe the baseline characteristics of ReDIRECT participants, focusing on socioeconomic and demographic factors as well as dietary and medical history, including LC diagnosis and symptoms.

## Methods

### Patient and Public Involvement

People living with Long COVID were involved throughout this research. Before submitting the funding application for the research, in March 2021, we conducted an online consultation via the Long Covid Scotland patient group (n=34) to assess interest in the study and to define priorities. Over 75% (n=26) were very interested, with 85% (n=30) prioritising the relief of fatigue and breathlessness as long COVID symptoms they would most like to improve. This informed our decision to use a novel personalised primary outcome measure.

The lay co-investigator (JO) has lived experience of Long COVID since March 2020 and is an active member of the Long Covid Scotland action group. JO and another co-author with lived experience of Long COVID (CW) were part of the study team and members of the monthly trial management team. A designated UoG staff member (TI) coordinated PPI input throughout, including input from a separate COVID-19 PPI group (6 people).

Throughout the study, the group regularly provided advice relating to the intervention, recruitment, data collection and topics for qualitative interviews, and also shaped dissemination activities, contributed to publications and the development of an animation aimed at the general public. Throughout the trial, the impact of involvement was tracked using the Guidance for Reporting Involvement of Patients and the Public’ (GRIPP2) short form, including highlighting where patient perspectives influenced study decision-making.

***Ethical approval*.** Ethical approval was obtained from the South-East Scotland Research Ethics Committee 01 (reference number: 21/SS/0077) on 19 ^th^ November 2021. The REC favourable opinion was subject to the following conditions being met prior to the start of the study.

Confirmation of Capacity and Capability (in England, Northern Ireland and Wales) or NHS management permission (in Scotland) should be sought from all NHS organisations involved in the study in accordance with NHS research governance arrangements. Each NHS organisation must confirm through the signing of agreements and/or other documents that it has given permission for the research to proceed (except where explicitly specified otherwise).

 At the time of writing, the latest version of the protocol was v1.3, dated June 15th, 2022 and the study status is ongoing, with the final participant visit scheduled in March 2024.

This trial was registered on ISRCTN registry ( ISRCTN12595520), on the 25th of November 2021. Study opened to recruitment on the 20th of December 2021.

***Study design and participants*.** The full protocol has been previously reported ( Haag *et al.*, 2022). In brief, ReDIRECT is a randomised, wait-list controlled, open-label study. The study is conducted entirely remotely, including recruitment, screening, written informed consent, randomisation, intervention, and outcome assessments.

The main inclusion criteria were people living with self-reported LC (symptoms persisting > 3 months before first recruitment contact, BMI > 27 kg/m ^2^ (BMI > 25 kg/m ^2^ for South Asians), and age ≥ 18 years. Study exclusion included lengthy hospitalisations (> 10 days) or intensive care unit (ICU) admissions related to COVID-19, people on insulin or anti-obesity drugs, proven myocardial infarction within the last 6 months, severe mental illness (including severe depression and eating disorder), women who are pregnant or considering pregnancy, history of substance abuse, active illness likely to cause a weight change, people who underwent bariatric surgery within the last 3 years or are planning bariatric surgery, advanced kidney problems (eGFR < 50 ml/min/1.73m ^2^), gallstones or pancreatitis, participating in another clinical research trial likely to affect diet or weight change, learning disability, and inability to understand English (written or spoken).

A total of 240 participants based in the UK were recruited through a wide range of avenues including social media (Facebook, Twitter), via support from individuals and organisations who disseminated the information within their networks (including Long Covid Scotland, Long Covid Podcast, GP practices, LC clinics, clinical research networks, councils and occupational health services across the UK, as well as colleagues in NHS Health Boards, Scottish government, and charities, including British Heart Foundation and Chest, Heart and Stroke Northern Ireland, The Wheatley Group, Men’s Shed Govan, Glasgow Weight Management Service, newspaper ads (Metro and Daily Mail), and local recruitment activities (e.g., posters and flyers at Glasgow libraries and leisure centres, local pharmacies, GP practices, supermarkets and banks, advert post on the University of Glasgow Yammer platform, community health promotion events at local places of worship, and local football clubs).

Research staff assessed eligibility during a screening telephone call, and participants gave informed consent electronically before enrolment.

After completing a baseline assessment, participants were randomised to either i) treatment: Counterweight-Plus programme (a professionally supported and evidence-based weight loss programme delivered entirely remotely), or ii) control: usual health care. The primary outcome is evaluated at the six-month time point, after which the control group gains delayed entry to the Counterweight-Plus intervention. Participants who did not complete the baseline assessment were withdrawn (n=5), and 235 were randomised. One participant withdrew consent following randomisation. In total, baseline data was available for 234 participants.

The published protocol describes the diet intervention ( Haag *et al.*, 2022). In brief, the Counterweight-Plus programme involves a very low-calorie total diet replacement (about 850 kcal/d) and behaviour change. The formula diet is consumed for 12 weeks, followed by food reintroduction and weight management up to the end of one year. Trained dietitians provide personalised support tailored to the needs of the individual via phone or video calls and text chats. Further support is provided through in-app weekly monitoring and nudges, personalised messaging, and group support.

***Outcomes and assessments*.** Outcomes were assessed for all participants at baseline prior to randomisation. Data were self-reported via bespoke online questionnaires, verbally over the telephone, email and/or text messages, and collected using a web-based bespoke electronic case report form (eCRF). The questions had pre-defined answer options (yes/no) and free text boxes for additional comments. The questionnaire can be found as *Extended data* to the published protocol ( Combet *et al.*, 2022). Participants received digital scales (Model UC-502, A&D Instruments Ltd, Abingdon, UK) and automatic blood pressure monitors (arm type monitor TMB-1970, Kinetik Medical Devices Ltd, Redhill, UK) for measurements of body weight and blood pressure. Self-reported outcomes included COVID-19 and LC-related outcomes, body composition measurements, comorbidities, prescribed medication, wellbeing and health outcomes, work productivity, healthcare use, and food expenditure Table 1.

**Table 1.** Primary and secondary outcome measures in the ReDIRECT trial. ( Haag *et al*., 2022). CFQ: Chalder Fatigue Scale, EQ-5D-5L: EuroQuol 5-dimensional 5-level questionnaire, LC: Long COVID, MRC: Medical Research Council.

	Primary outcome measures	Scales / Tools	References
Self-selected LC symptom	Fatigue	Validated Chalder Fatigue Scale CFQ-11	( Chalder *et al*., 1993)
	Breathlessness	Modified MRC Dyspnoea Scale	( Bestall *et al*., 1999)
	Pain	P4 Numeric Pain Rating Scale	( Spadoni *et al*., 2004)
	Anxiety and depression	Hospital Anxiety and Depression Scale (HADS) questionnaire	( Zigmond & Snaith, 1983)
	Other	Visual Analogue Scale (0-10) for other symptoms with no pre-specified scale	
	Secondary outcome measures	Scales / Tools	References
All non-selected primary LC symptoms	Fatigue, breathlessness, pain, anxiety/depression, other	As above	As above
Body composition and health	Weight	Digital scales (Model UC-502, A&D Instruments Ltd, Abingdon, UK)	
	Height		
	Blood pressure	Arm type monitor (TMB-1970, Kinetik Medical Devices Ltd, Redhill, UK)	
	COVID vaccination		
	Medical history		
	Medication		
Other	Quality of life	EQ-5D 5L	( Buchholz *et al*., 2018)
	Work productivity	Work Productivity and Activity Impairment	( Reilly *et al*., 1993)
	Healthcare utilisation	Bespoke questionnaire	
	Food expenditure	Bespoke questionnaire	

### LC symptoms

LC symptoms were assessed using validated questionnaires for the four core symptoms of fatigue (Chalder Fatigue Scale, CFS ( Chalder *et al.*, 1993)), breathlessness (modified Medical Research Council dyspnoea scale mMRC ( Bestall *et al.*, 1999)), pain (P4 pain scale ( Spadoni *et al*., 2004)) and anxiety/depression (Hospital Anxiety and Depression Scale, HADS ( Zigmond & Snaith, 1983)). Caseness for the four core symptoms was determined as follows.

The CFS is an 11-item scale with four answer options: 'less than usual', 'no more than usual', 'more than usual', and 'much more than usual'. A bimodal scoring system for each item (0, 0, 1, 1) was used and summed to a total score ranging from 0 to 11. As established in the validation study ( Chalder *et al.*, 1993), a score of at least 4 was used to indicate the caseness of fatigue. A continuous score (0, 1, 2, 3) can also be used to assess the intensity of symptoms. For both scoring systems, a higher score indicates greater fatigue severity. The CFS can be subdivided into two components to separately measure physical fatigue (items 1 to 7) and mental fatigue (items 8 to 11).

The HADS questionnaire comprises two subscales for anxiety and depression, with seven items, each scored 0 to 3. A total sum of 11 or more for each subscale was used to indicate the caseness of anxiety or depression ( Zigmond & Snaith, 1983).

The P4 pain scale consists of four items which assess pain levels on a numeric scale of 0 to 10 in the morning, afternoon, and evening, and with activity with a total score ranging from 0 (no pain) to 40 (pain as bad as it can be). A caseness for pain was considered with a score of 1 or more.

The mMRC is a tool to assess the degree of breathlessness on a scale of 0 (no breathlessness) to 4 (extreme breathlessness). A score of at least 1 ('I get short of breath when hurrying on level ground or walking up a slight hill' or worse) indicated breathlessness.

Participants could add further LC symptoms in free text boxes along with a rating on how troublesome the symptoms were on a 10-point numeric scale. All symptoms entered at baseline were considered active.

### Health status

Health status on the day of the baseline assessment was measured using the EQ-5D-5L questionnaire ( Buchholz *et al.*, 2018). This validated tool indicates health status across five dimensions for mobility, self-care, usual activities, pain/discomfort and anxiety/depression with five levels each (no, slight, moderate, severe or extreme problems). The combination of these five domains can be mapped to a health utility score, which ranges from 0 (a state as bad as being dead) to 1 (perfect health) ( Hernandez Alava *et al.*, 2023). An additional visual analogue scale asks the participant to rate their health on the day, ranging from 0 ('The worst health you can imagine') to 100 ('The best health you can imagine'). Data were analysed by individually considering each dimension and the VAS and combining the five dimensions to a composite health status.

***Statistical analysis*.** Baseline data is presented as n (%) for categorical variables and mean (SD) or median (IQR) for continuous variables. Percentages for recruitment are based on the total number of participants recruited (n=240). All other percentages are based on the available data (n=234).

Free text responses were evaluated and grouped into categories using Microsoft Excel following a defined coding dictionary (see extended data). Free text data and pre-defined answer options were combined where they overlapped to avoid duplication.

Medication entries were analysed as medications ( Table 3) if included in the British National Formulary (BNF) catalogue and grouped following the main BNF categories. Entries not considered in the medication analysis (n=15) include Androfeme, Sterimar nasal spray, several dietary supplements (multivitamins, vitamin C, cod liver oil, flushing niacin, quercetin, co-enzyme Q10, probiotics, blackcurrant seed oil, flaxseed oil, bromelain), and CBD oil. All dietary supplements entered as medications were also included in the nutritional supplement analysis ( Figure 6H). CBD oil was included in private healthcare.

Group differences between those who had COVID-19 before and after vaccination were tested using the Mann-Whitney-U test in R for the median number of symptoms and the chi-squared test for the type of symptoms. A p-value of 0.05 was considered statistically significant.

All graphs were created in R using the ggplot2 package and HH package. Plot panels were compiled using Inkscape. BioRender.com was used to partially created one of the figures.

## Results

### Study population

In total, 240 participants from across the UK were enrolled in the ReDIRECT study, of whom 235 (98%) were randomised to either the diet intervention Counterweight-Plus (n=117, treatment group) or usual care (n=118, control group). One participant in the treatment group withdrew consent after randomisation, resulting in n=116 in the treatment group.

The main source of recruitment was social media (n=104, 43%), followed by other online sources, including e-newsletters, websites, and podcasts (n=40, 17%), communication through health care professionals (n=33, 14%), word of mouth (n=31, 13%), LC support groups (n=13, 5%), and newspapers (n=8, 3%). Local recruitment activities (at Glasgow Gurdwaras and football clubs) and GP practice searches conducted in 14 practices in the NHS Greater Glasgow and Clyde health board (32 invitations sent to participants) were least successful with only n=4 (2%) and n=3 (1%) recruited, respectively. One person in the Public Patient Involvement group participated; the recruitment source was unknown for 3 participants. These methods facilitated rapid recruitment of 240 participants in slightly over 6 months.

Baseline demographic characteristics are described in Table 2. Most participants were women (n=198, 85%) of white ethnicity (n=211, 90%) from a range of socioeconomic backgrounds with a mean age of 46 years (SD 10), a median BMI of 35 kg/m ^2^ (IQR 32 - 40) and at least an undergraduate degree (n=143, 61%). Participants lived in England (n=149, 64%), Scotland (n=71, 30%), Wales (n=11, 5%), and Northern Ireland (n=3, 1%). While participants were from across the spectrum of area deprivation, those living in the most deprived areas were somewhat less represented than those from the least deprived areas (13% vs. 27%). Most participants reported living with others in a household (n=204, 87%). Most lived with a partner/spouse (n=167, 82%) and/or at least one child (n=127, 54%).

**Table 2.** Participant characteristics at baseline.

Characteristics (n % unless otherwise specified)	
n	234
Women	198 (85%)
Age, years, mean (SD)	46 (10)
Systolic blood pressure, mmHg, mean (SD)	131 (14)
Diastolic blood pressure, mmHg, mean (SD)	80 (9)
Body mass index, kg/m ^2^ (IQR), n (%)	35 (32 - 40)
Below 27 (South Asian ethnicity)	1 (<1%)
27 to 29.9	32 (14%)
30 to 34.9	80 (34%)
35 to 39.9	58 (25%)
40 or above	63 (27%)
Region	
England	149 (64%)
Scotland	71 (30%)
Wales	11 (5%)
Northern Ireland	3 (1%)
Ethnicity	
White	211 (90%)
South Asian	10 (4%)
Other Asian, Asian British	5 (2%)
Black, African, Caribbean or Black British	2 (1%)
Other or mixed ethnic group	6 (3%)
Education	
School leaver/standard grade/GCSE	28 (12%)
Highers/A levels	14 (6%)
Higher education HND/HNC/NVQs	47 (20%)
Undergraduate degree	87 (37%)
Master’s degree/PhD	56 (24%)
Prefer not to say	2 (1%)
Living situation	
Alone	30 (13%)
With partner	167 (71%)
With children under 18	106 (45%)
With children over 18	29 (12%)
With parents	16 (7%)
With siblings	15 (6%)
With friends	4 (2%)
Other (parents-in-law, grandparents, lodger)	3 (1%)
Index of multiple deprivation	
1 (most deprived)	31 (13%)
2	44 (19%)
3	45 (19%)
4	51 (22%)
5 (least deprived)	63 (27%)

### Employment

**Figure 1.**
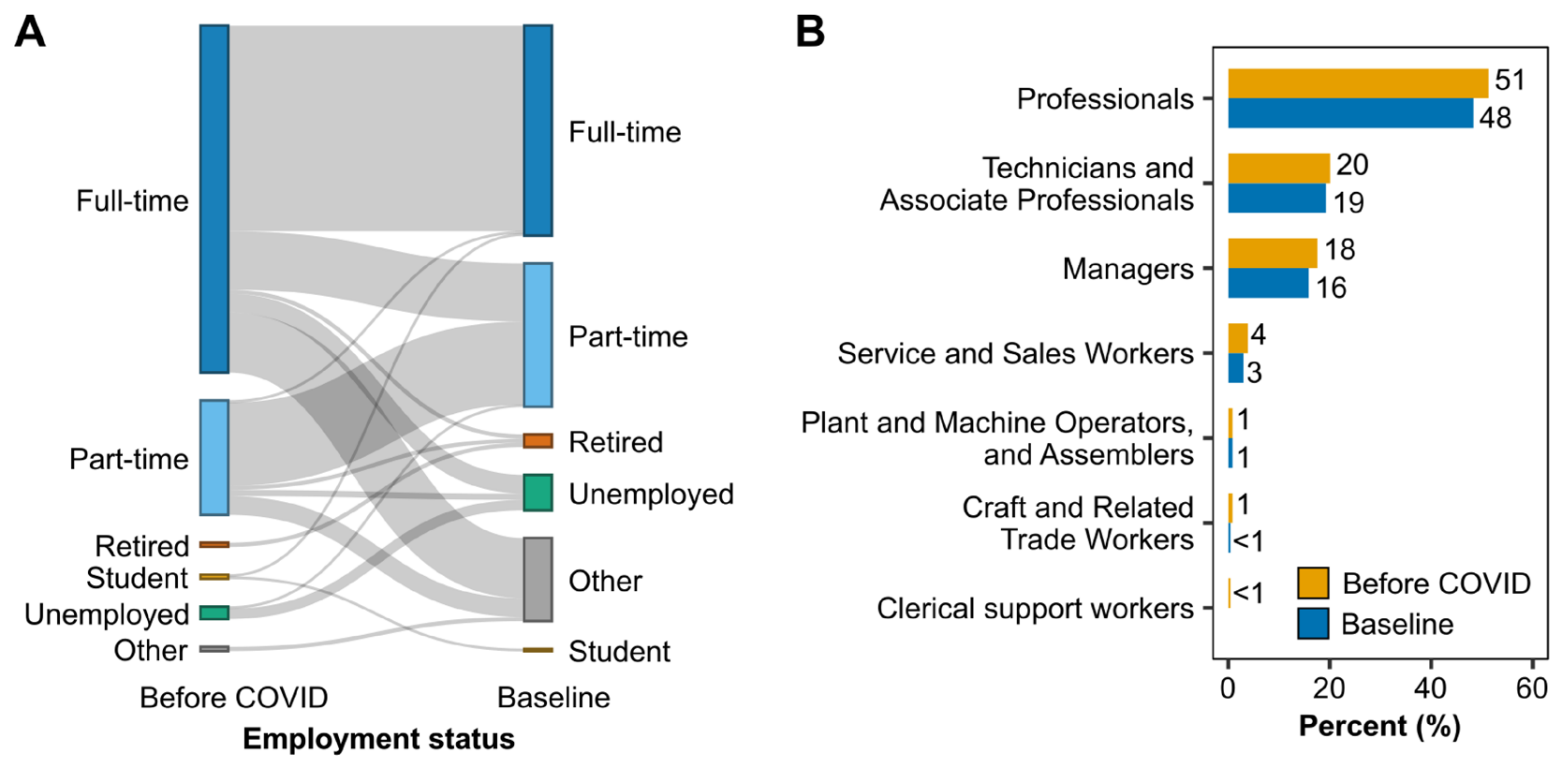
Employment status and occupation groups before COVID and at baseline. ( **A**) Participants were asked to choose one of the six categories in the figure which best described their employment status before COVID-19 and at baseline. Pre-covid, ‘other’ includes homemaker and full-time carer/part-time student. At baseline, ‘other’ includes long-term sick leave, homemaker, disabled, part-time student, and agency worker. ( **B**) Occupations were classified based on the International Standard Classification of Occupations (ISCO-08) and ranged across seven of the ten major occupational groups both before COVID-19 and at study baseline.

Participants were asked to choose one of the six categories that best described their employment status before COVID-19 and at baseline (full-time, part-time, retired, student, unemployed, other). Between becoming ill with COVID-19 and entering the study, 85 (36%) participants experienced a change in employment status ( Figure 1A). Before the first COVID-19 infection, most participants (n=167, 71%) were in full-time employment, compared to 101 (43%) at the start of the study. Following COVID, changes in full-time employment included switching to part-time (n=28, 12%), being on long-term sick leave (n=28, 12%), reduced working hours (n=6, 3%), retiring (n=2, 1%), or becoming unemployed (n=9, 4%). Including people who worked part-time, 44 (19%) participants were on long-term sick leave, and 7 (3%) were on reduced hours due to LC at baseline.

Based on the International Standard Classification of Occupations (ISCO-08), occupations ranged across seven major occupational groups ( Figure 1B). Healthcare and teaching professionals were the most common occupation groups, with over one-third of participants working in healthcare and over 10% in the educational sector. No participant worked as a skilled agricultural, forestry and fishery worker in elementary occupations or the armed forces.

### Contracting COVID-19

**Figure 2.**
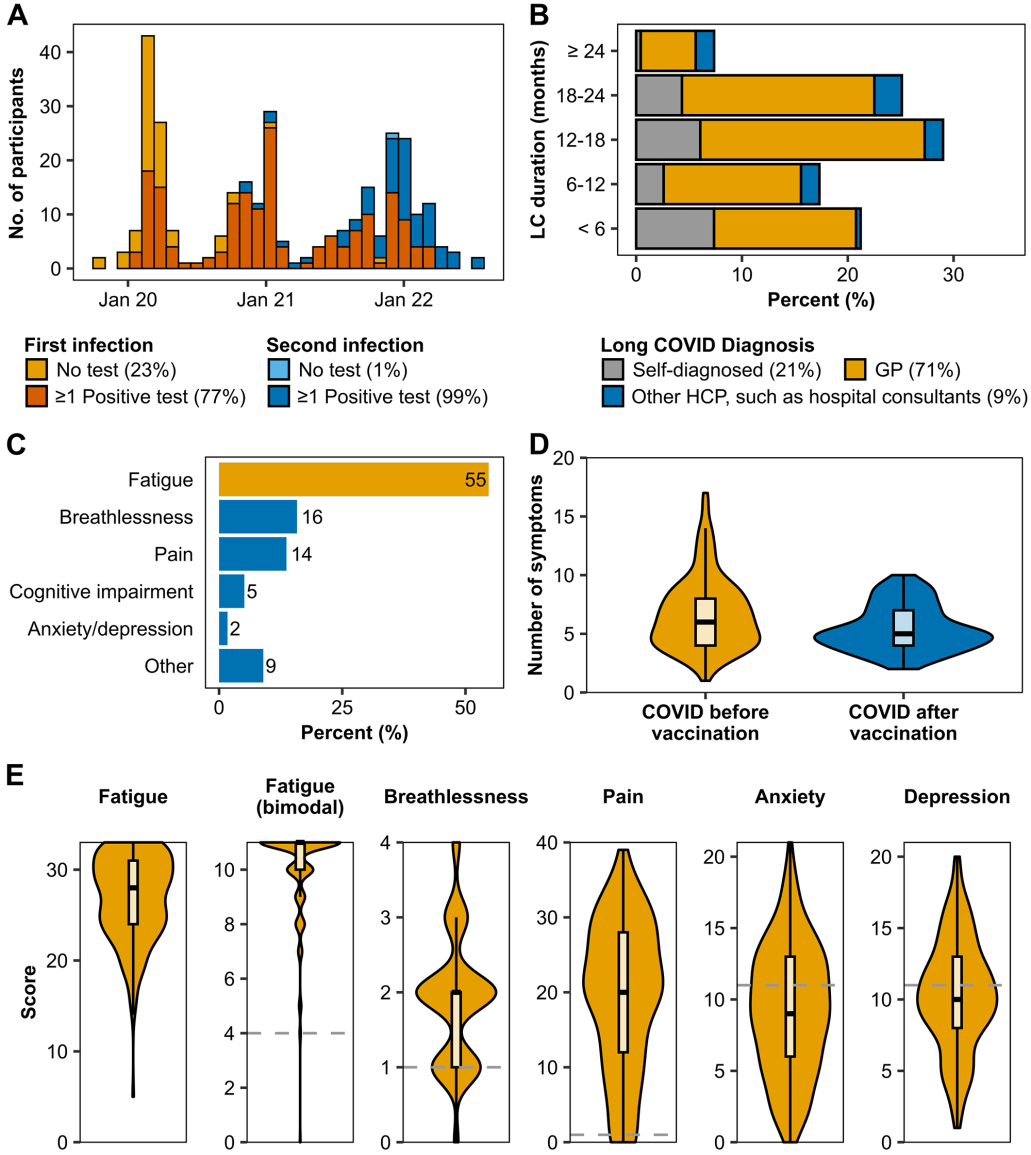
Covid-19 infection and Long COVID symptoms. ( **A**) COVID-19 infection dates: First COVID infection dates ranged from October 2019 to March 2022, and second infection dates ranged from November 2020 to July 2022. Most infections were confirmed by at least one positive COVID test (PCR, rapid antigen lateral flow test, or antibody test), shown in orange and dark blue. Yellow and light blue bars show the number of infections which were not confirmed by a COVID test. ( **B**) Long COVID duration: Figure shows the length of time in months participants were living with Long COVID at the start of the study. Long COVID was diagnosed either by a GP (yellow), a different healthcare professional (blue), or self-diagnosed (grey). GP: general practitioner, HCP: healthcare professional, LC: Long COVID. ( **C**) Percentage of the Long COVID symptom each participant selected as the one they would most like to see improve. The most selected symptom (fatigue) is highlighted in yellow. ( **D**) Number of Long COVID symptoms (core symptoms and symptom groups) in people who had COVID before (yellow) and after (blue) vaccination. Difference between the groups was significant (p=0.003). ( **E**) Core Long COVID symptoms: Fatigue, breathlessness, pain, anxiety, and depression were assessed using validated questionnaires (Chalder Fatigue Scale, modified MRC Dyspnoea Scale, P4 pain scale, Hospital Anxiety and Depression Scale). Fatigue is represented as total fatigue score and bimodal scoring. The y-axes show complete range of possible scores for each scale (0 to 33, 11, 4, 40, 21, 21 for fatigue total, fatigue bimodal, breathlessness, pain, anxiety, and depression, respectively), with higher scores indicating greater severity. The grey dashed line shows the cutoffs used for caseness of the symptoms. No cutoff for total fatigue score was used to determine caseness.

Dates of the first COVID-19 infection ranged from October 2019 to March 2022 ( Figure 2A). Most participants (n=178, 76%) had at least one positive COVID-19 test (PCR 64%, LFT 37%, antibody test 19%, unsure which test 2%). Those who did not have a positive COVID-19 test predominantly became ill in the first five months of the outbreak (up to May 2020). Following the first infection, 30 (13%) were admitted to the hospital (mean length of stay 6 days (SD 5)).

A third of participants (n=71, 30%) contracted COVID-19 at least a second time before the start of the study, which mostly occurred in the winter wave in 2021/2022 ( Figure 2A). Nearly all (n=70, 99%) had at least one positive test (PCR 68%, LFT 77%, antibody test 7%). Hospital admission rates following the second infection were slightly lower than the first (n=5, 7%), and the mean stay was 3 days (SD 3).

At the start of the study, most participants (n=228, 97%) had been vaccinated at least once, with 2 (1%) having received one vaccination, 19 (8%) two vaccinations, and 207 (91%) three vaccinations. Most participants contracted COVID-19 for the first time before their first vaccination (n=169, 72%). Of the 65 participants who contracted COVID-19 after the first vaccination, 8 (3%) became ill before the second vaccination, 28 (12%) became ill after the second but before the third vaccination, and 26 (11%) became ill after the third vaccination.

Data on any further infections prior to the study start were not collected.

### Long COVID

LC was mainly diagnosed by a GP (n=166, 71%), other healthcare professionals such as hospital consultants (n=20, 9%) or self-diagnosed (n=48, 21%) ( Figure 2B). At baseline, most participants had lived with LC for at least one year (n=142, 61%). A small proportion reported having had LC over two years (n=17, 7%).

***Symptoms*.** The dominant LC symptom was fatigue (n=128, 55%), followed by breathlessness (n=37, 16%), pain (n=32, 14%), and cognitive impairment (n=12, 5%) ( Figure 2C). Outside of these four symptoms, 25 participants (11%) named symptoms such as anxiety/depression, loss of taste and smell, tremors, and tinnitus as their main symptom.

Fatigue was prevalent in all but one participant (n=233, 99.6%) (assessed using the bimodal scoring system) ( Figure 2E). The continuous scores ranged from 0 to 33, with a high median score of 28 (IQR 24 – 31). The median physical fatigue score was 18 (IQR 15 – 20, max 21), while the median mental fatigue score was 10 (IQR 8 – 12, max 12).

Similarly, both breathlessness (n=228, 97%) and pain (n=219, 94%) were highly prevalent among participants ( Figure 2E). The median for breathlessness was 2 (IQR 1 – 2) of a maximum of 4, i.e. most participants had moderate breathing difficulties ('On level ground, I walk slower than people of the same age because of breathlessness or have to stop for breath when walking at my own pace').

Pain severity was spread across the entire range from no pain at all (score 0) to pain as bad as it could be (score 40) ( Figure 2E). The median for pain was 20 (IQR 12 – 28).

Anxiety (n=95, 41%) and depression (n=110, 47%) were both prevalent in less than half of participants ( Figure 2E). The median score for anxiety was 9 (IQR 6-13), and the median for depression was 10 (IQR 8-13) out of a maximum of 21 points for both symptoms.

**Figure 3.**
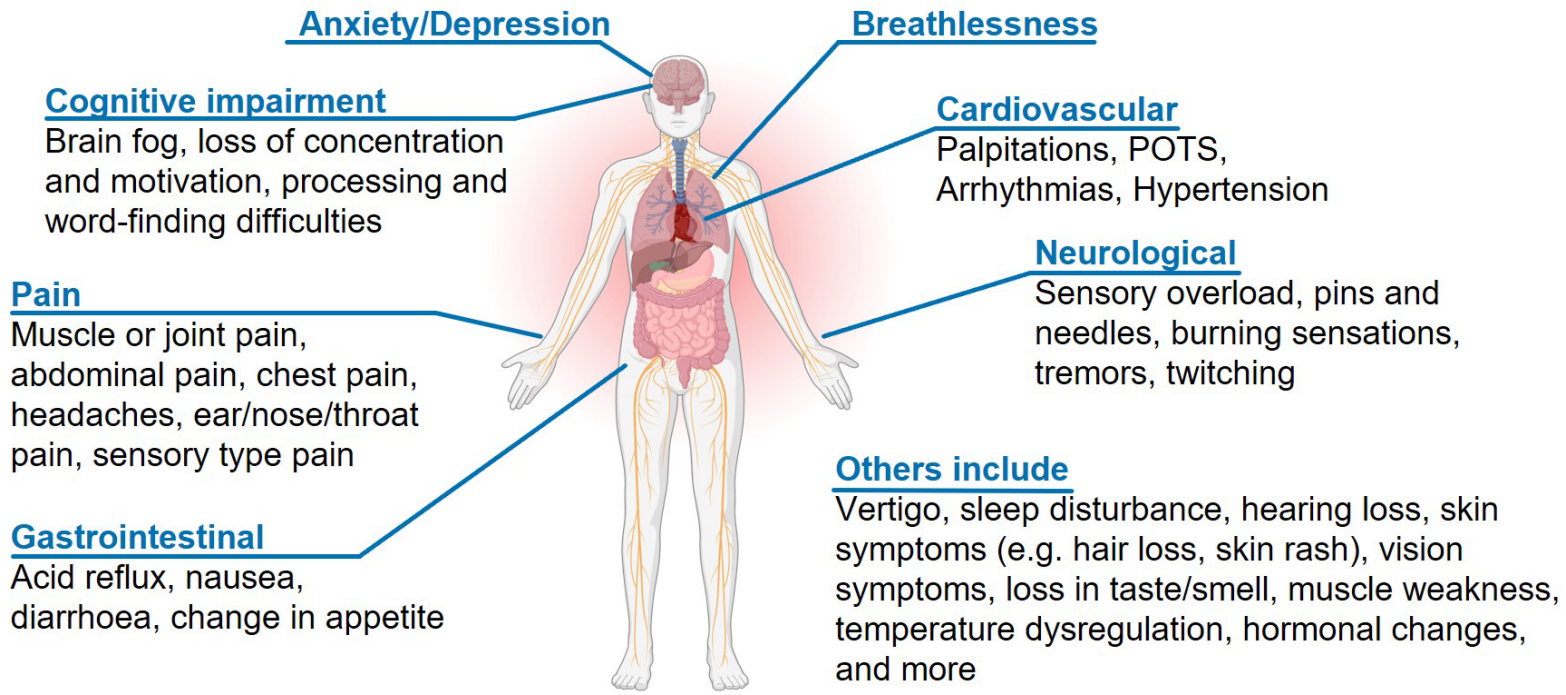
Overview of Long COVID symptoms reported in the ReDIRECT study. Participants reported Long COVID symptoms outside of fatigue, breathlessness, pain, and anxiety/depression using free text boxes. Partially created with Biorender.com.

Aside from the core symptoms (fatigue, breathlessness, pain, anxiety/depression), 179 (76%) participants reported other symptoms, grouped into 27 symptom groups. Symptom groups experienced ranged from 0 to 13 (median 2 (IQR 0 – 4), related to cognitive impairment (n=82, 35%), neurological symptoms such as tremors and feelings of pins and needles (n=66, 28%), cardiovascular symptoms such as palpitations and arrhythmias (n=52, 22%), and digestive disorders including acid reflux, nausea and diarrhoea (n=51, 22%). Figure 3 shows an overview of reported LC symptoms.

The median total number of symptoms was 6 (IQR 4 – 8). The number of symptoms experienced by people who fell ill with COVID-19 before their first vaccination (n=163) and those who got COVID-19 after vaccination (n=65) are displayed in Figure 2D. People who contracted COVID-19 before vaccination had a median of 6 symptoms in comparison to 5 (p=0.003), with more frequent reports of neurological symptoms (34% vs 14%, p=0.004), cutaneous signs (18% vs 3%, p=0.005), hearing loss or tinnitus (20% vs 6%, 0.021), and digestive disorders (25% vs 12%, p=0.045) (Supplementary Figure 2, which can be found as *Extended data* ( Haag *et al.*, 2023b)).

***Support*.** Most participants received support from the NHS to manage LC (n=206, 88%). Most participants visited their GPs (n=190, 81%), and half visited specialist LC clinics (n=117, 50%). Hospital specialist services were used by n=113 (48%), and a third (n=74, 32%) received physiotherapy. Only a small proportion received dietary support (n=19, 8%) or mental health support (n=11, 5%). A minority said they received no support (n=29, 12%). In addition to NHS resources, 56 (24%) paid for private healthcare, and 16 (7%) used complementary and alternative medicine. Private healthcare included medical specialists (n=24, 10%), Nuffield Health (n=15, 6%), hyperbaric oxygen treatment (n=8, 3%), physiotherapy (n=7, 3%), nutritionist (n=4, 2%), mental health support (n=3, 1%), private GP (n=2, 1%), medication and testing (n=1, <1%), and LC rehab (n=1, <1%).

### Comorbidities, medications, health status

**Figure 4.**
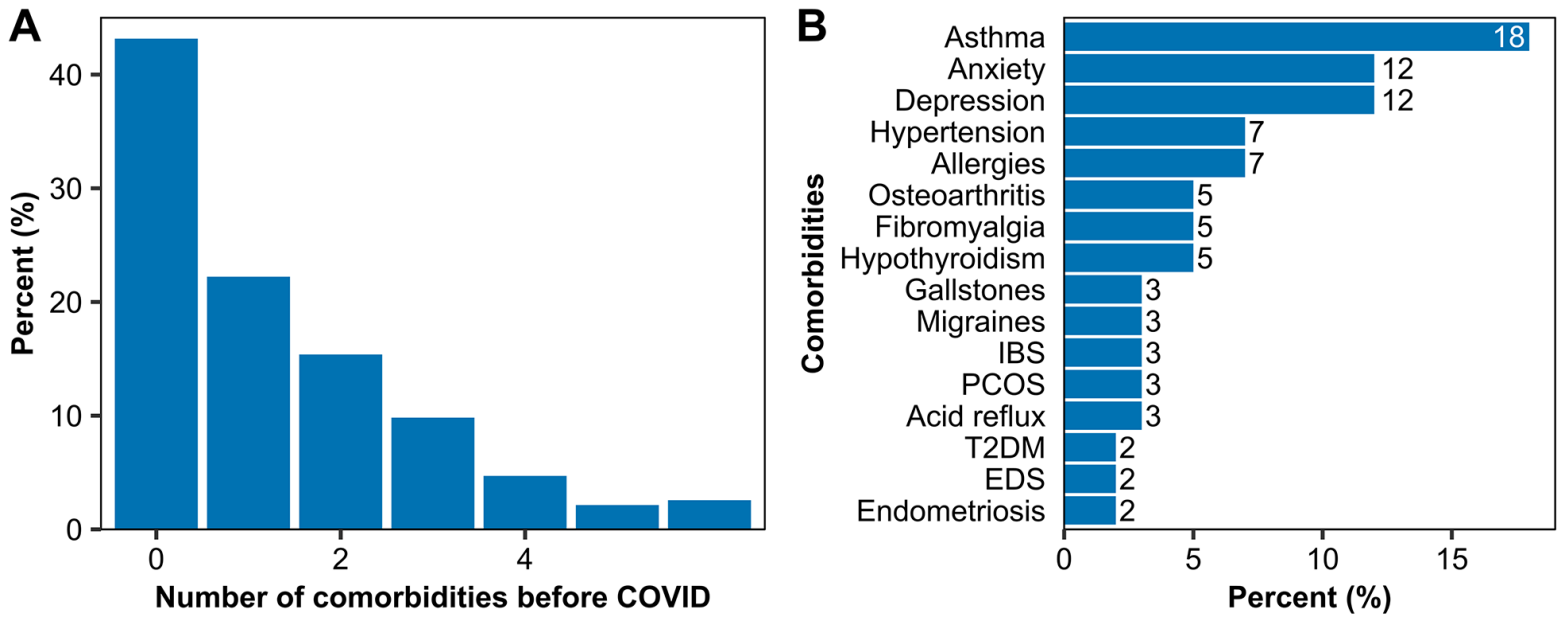
Existing comorbidities prior to the first COVID infection. ( **A**) The number of comorbidities per participant and ( **B**) the 16 most prevalent comorbidities which were present before the first COVID infection.

At baseline, the median weight was 98 kg (IQR 86 – 114), and the median BMI was 35.2 kg/m ^2 ^(IQR 32 – 40). Mean blood pressure was 131 (SD 14) / 80 (SD 9) mmHg. Over half of the participants (n=133, 57%) reported at least one other medical condition diagnosed before contracting COVID-19 ( Figure 4 and Supplementary Table 6, which can be found as *Extended data* ( Haag *et al.*, 2023b)).

The median number of comorbidities was 1 (IQR 0-2), and the total ranged from 0 to 12, not counting overweight and obesity. The most prevalent comorbidity was asthma (n=42, 18%), followed by anxiety (n=29, 12%), depression (n=28, 12%), and hypertension (n=17, 7%). Prior to COVID-19, 12 participants (5%) reported fibromyalgia, 5 (2%) type 2 diabetes mellitus, and 4 (2%) ME/CFS. A full list of comorbidities prior to COVID-19 and their frequency can be found in Supplementary Table 7 as *Extended data* ( Haag *et al.*, 2023b).

At baseline, most participants (n=191, 82%) were taking medication, with the total number of medications per participant ranging from 1 to 16 (median (IQR) 4 (2 – 6)). Over half of the participants had been prescribed at least one medication to treat symptoms of LC (n = 124, 53%), while 28 (12%) took at least one medication for which they reported not knowing whether they were taking it for LC symptoms or a different condition.

In total, 818 different medications were reported ( Table 3), of which 42% were taken to treat LC and 51% were prescribed for other health conditions. The treated condition was unknown to the participant for 6% of medications.

**Table 3.** Medications. Frequency and type of medications taken at baseline.

Drug group	n (%) of n=818 medications	n (%) of n=234 participants
Central Nervous System	235 (29%)	125 (53%)
Respiratory System	163 (20%)	89 (38%)
Gastro-Intestinal System	95 (12%)	79 (34%)
Cardiovascular System	118 (14%)	73 (31%)
Obstetrics, Gynaecology and Urinary-Tract Disorders	51 (6%)	41 (18%)
Endocrine System	42 (5%)	33 (14%)
Nutrition and Blood	51 (6%)	32 (14%)
Musculoskeletal and Joint Diseases	32 (4%)	29 (12%)
Ear, Nose and Oropharynx	10 (1%)	10 (4%)
Infections	7 (1%)	6 (3%)
Malignant Disease and Immunosuppression	4 (<1%)	3 (1%)
Skin	3 (<1%)	3 (1%)
Eye	1 (<1%)	1 (<1%)

Common medications included drugs for the central nervous system (n= 125, 53% of participants), respiratory system (n=89, 38% of participants), gastro-intestinal system (n=79, 34% of participants), cardiovascular system (73, 31% of participants) and obstetrics, gynaecology, and urinary tract disorders (n=41, 18% of participants).

A full list of medications can be found in the supplementary data as *Extended data* ( Haag *et al.*, 2023b).

**Figure 5.**
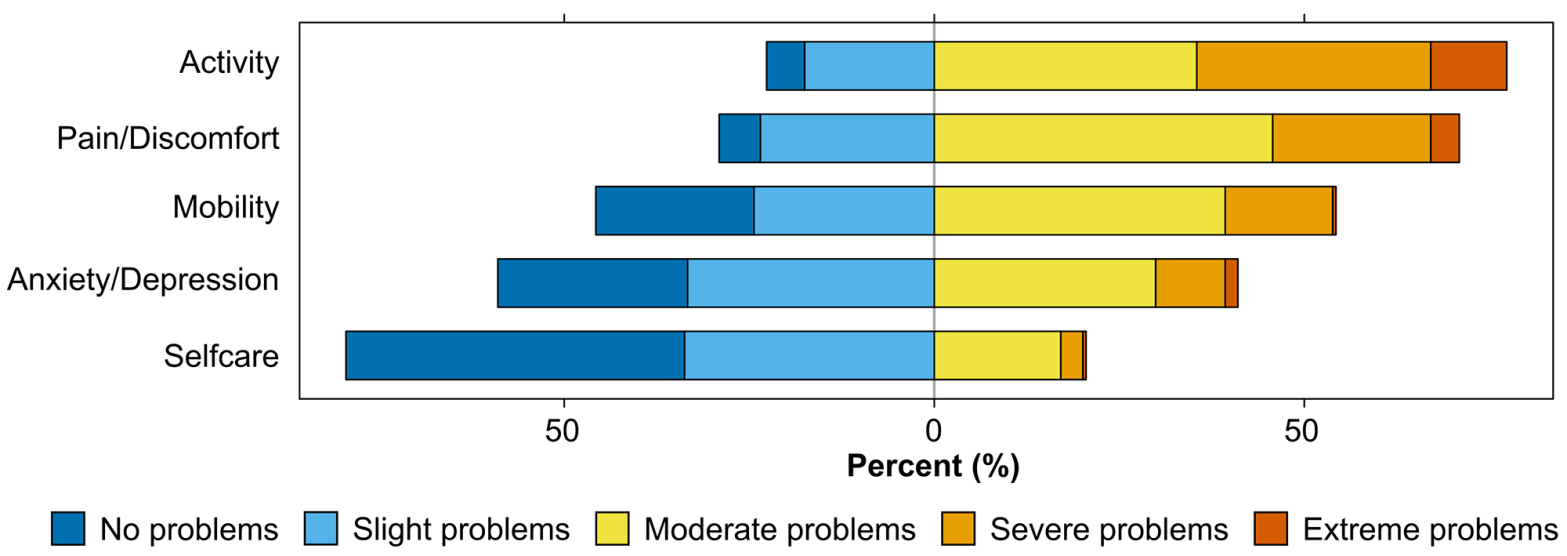
Health status at baseline. Health status was assessed using the EQ-5D-5L questionnaire across five dimensions (activity, pain/discomfort, mobility, anxiety/depression, selfcare) and five levels each (no, slight, moderate, severe, or extreme problems). All participants (n=234) responded to the questionnaire.

Health status was measured using the EQ-5D-5L questionnaire ( Figure 5). At baseline, most participants reported moderate or worse problems with activity (n=181, 77%), pain/discomfort (n=166, 71%), and mobility (n=127, 54%). Slightly less than half reported moderate or worse problems with anxiety/depression (n=96, 41%). Participants had the least problems with self-care (washing or dressing themselves) (moderate or worse problems: n=48, 20%). The overall health utility score (combining all five domains) was mean (SD) 0.48 (0.243). The mean EQ-5D VAS was 46 (SD 17) (Supplementary Table 9 ( Haag *et al.*, 2023b)).

### Support networks

LC support networks, such as online communities, were used by 139 (59%) participants (Supplementary Table 11 ( Haag *et al.*, 2023b)). The most common networks included Facebook groups (n=98, 42%) and LC support charities (n=58, 25%; includes Long Covid Support, Long Covid Scotland, Long Covid England, Long Covid Wales, Long Covid SOS). Further support was accessed through local support groups (n=18, 8%), online forums and communities such as Twitter and Reddit (n=12, 5%), and WhatsApp groups (n=5, 2%).

### Weight management and diet

Most participants (n=184, 79%) provided pre-COVID-19 weight data: median weight before COVID-19 was 84.4 kg (IQR 73.5 – 97.2) and 98.0 kg (IQR 86.3 – 114.0) at baseline.

**Figure 6.**
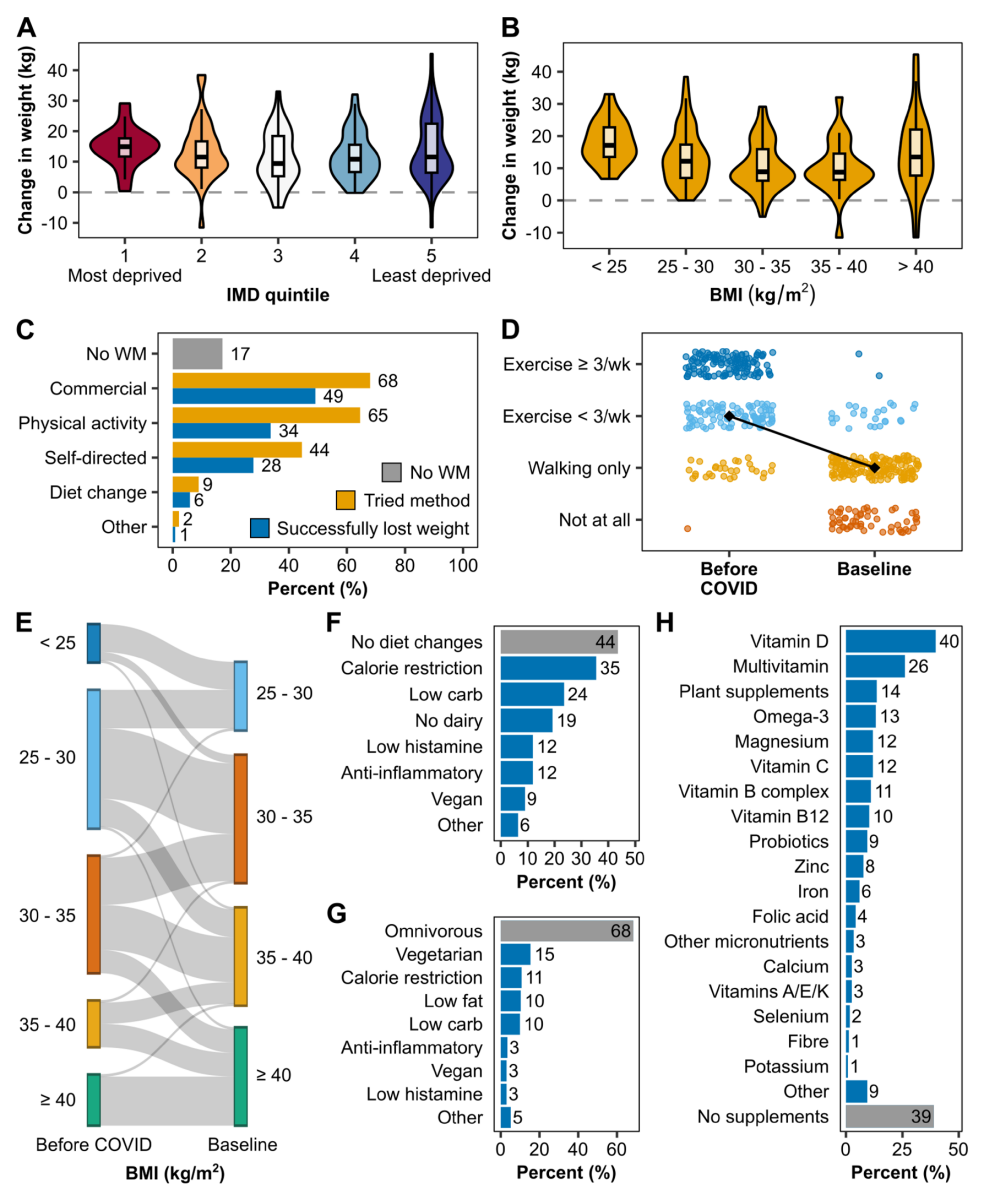
Weight management, diet and lifestyle changes from before COVID to baseline. Pre-COVID weight data was provided by 184 (79%) participants. The change in weight from before the first COVID infection to the baseline assessment is shown per IMD quintile ( **A**) and BMI category prior to COVID ( **B**). BMI (kg/m ^2^) was categorised as follows: < 25 = normal weight, 25 – 30 = overweight, 30 – 35 = obesity class I, 35 – 40 = obesity class II, > 40 = obesity class III. The number of participants per BMI category before COVID and how their weight status had changed at baseline is illustrated in ( **E**). ( **C**) Most participants had prior experience with weight management using different methods (yellow) with varying degrees of success in losing weight (blue). A minority did not attempt to lose weight prior to joining the study (grey). ( **D**) Physical activity levels before COVID and at baseline were reported in four levels: regular exercise (≥ 3 times per week), occasional exercise (< 3 times per week), walking only, or no physical activity at all. The black diamond and line show the change in median activity levels before and after COVID. ( **F**) Dietary changes following the development of Long COVID were common. Other diets not explicitly listed include reduction in lactose, gluten, FODMAPs, sugar, as well as vegetarian, pescetarian, anti-obesity medication, and general healthy eating. ( **G**) shows the diet consumed at baseline. Other includes reduction in gluten, dairy, lactose, sugar, FODMAPs and increase in healthy eating. Supplements taken at baseline are listed in ( **H**). For the full list of supplements, see Supplementary Table 16, which can be found as *Extended data* ( Haag *et al.*, 2023b). BMI: body mass index, IMD: index of multiple deprivation, WM: weight management.

Following COVID, high weight gain was prevalent across all areas of deprivation ( Figure 6A). Over 90% of participants reported weight gain (n=215, 92%). A few participants reported not changing in weight (n=10, 4%), losing weight (n=6, 3%), or did not know how their weight changed in comparison to before COVID-19 (n=3, 1%). The highest reported weight gain was 45.3 kg, and the highest weight loss was 11.5 kg. In total, the median (IQR) change in weight from before COVID-19 to the study baseline was 11.5 kg (6.9 – 16.7).

Weight status NICE weight classes ( https://cks.nice.org.uk/topics/obesity/diagnosis/identification-classification/) changed for 65 (28%) participants. In most cases (n=47, 20%), weight status changed from overweight to obesity classes. Of the 18 participants who were of normal weight before COVID-19, their weight status changed to overweight (n=13, 6%) or obesity (n=5, 2%) ( Figure 6E).

We found that median weight gain was highest for people with a pre-COVID BMI below 25 (n=18, 17.1 kg (OQR 13-23)) and lowest for people with a pre-COVID BMI of 35-40 (n=22, 8.8kg (IQR 6-15)) ( Figure 6B, Supplementary Table 13 ( Haag *et al.*, 2023b)). Weight gain was similar across IMD quintiles ( Figure 6A).

Before starting the study, most participants already had experience with weight management (n=194, 83%), of which 80% had successfully lost weight in the past ( Figure 6C). Commercial weight loss programmes were the most used method (n=159, 68%) and the most successful for weight loss (n=115, 72% of those who used commercial weight management). Other methods included increased physical activity, self-directed weight loss programmes (e.g., the 5:2 diet), diet change and other methods (bariatric surgery and practising meditation). Pre-COVID-19 BMI and BMI at baseline were higher in those with prior weight management experience (median pre-COVID BMI: 29.2 vs 25.6 kg/m ^2^, median BMI at baseline: 35.6 vs 32.6 kg/m ^2^). The median change in weight after COVID-19 was similar regardless of prior weight management experience (8.4 vs 8.2 kg).

Lifestyle changes were common after COVID. Notably, physical activity was reduced for 9 out of 10 participants (n=212, 91%) ( Figure 6D). On average, median physical activity levels reduced from exercising occasionally (< 3 times per week) to walking only. The number of people who did not exercise at all rose from n=1 (<1%) before COVID-19 to n=54 (23%) at baseline.

Over half of the participants (n=132, 56%) modified their diet after developing LC, with calorie restriction (n=85, 36%) and low-carb diets (n=56, 24%) being the most common, followed by no dairy (n=49, 21%), low histamine (n=33, 14%), anti-inflammatory (n=32, 14%), vegan (n=21, 9%), and other (such as gluten-free and low FODMAP) (n=9, 4%) ( Figure 6F). A third tried more than one type of diet (n=76, 32%). At baseline, most participants were following an omnivorous diet (n=160, 68%) and n=143 (61%) were taking supplements ( Figures 6G and 6H). The most common three supplements were Vitamin D (n=93, 40%), multivitamins (n=61, 26%), and plant supplements and bioactives (n=32, 14%).

Further lifestyle changes included stopping drinking alcohol (n=41, 21%) and stopping smoking (n=7, 9%).

## Discussion

### Summary of key findings

The baseline characteristics of participants in the Remote Diet Intervention to Reduce Long-COVID Symptoms Trial (ReDIRECT) describe a diverse group of adults across the UK who are predominantly middle-aged women of white ethnicity.

The remote nature of the study allowed for rapid recruitment, with a broad geographic spread of participants across the UK. While participants were from a range of socioeconomic backgrounds, those living in the most deprived areas were underrepresented compared to those from the least deprived areas (13% vs. 27%). Two-thirds of participants had at least an undergraduate degree, with a third working in healthcare. Representation of healthcare professionals reflects some of our recruitment strategies, including dissemination through NHS Health Boards in Scotland and electronic bulletins aimed at healthcare professionals, and the fact that front-line staff were markedly more exposed to COVID-19 in the earlier parts of the pandemic ( Nguyen *et al.*, 2020).

A critical methodological feature of the ReDIRECT study is the personalised primary outcome, with participants nominating at baseline the LC symptom that they would most like to see improve ( Haag *et al.*, 2022). The LC symptom nominated by most participants was fatigue, followed by breathlessness, pain, and cognitive impairment. A small proportion of participants named other symptoms (such as anxiety/depression, loss of taste and smell, tremors, and tinnitus) as their main symptom. These symptoms have also commonly been reported in other LC studies ( Davis *et al.*, 2021; Kim *et al.*, 2023; Lopez-Leon *et al.*, 2021; Marjenberg *et al.*, 2023; Sudre *et al.*, 2021; Whitaker *et al.*, 2022).

Fatigue was reported by nearly all participants, with a high median score on the Chalder Fatigue Scale. Similarly, both breathlessness and pain were highly prevalent, affecting close to all participants. Anxiety and depression were present in approximately half of the participants, reflecting a high level of illness burden and distress across the study population, with corresponding impacts on quality of life. There is a well-established relationship between obesity and health-related quality of life. However, the EQ-5D VAS mean score of 46 (SD 17) in the ReDIRECT study population is markedly lower than would be expected from raised BMI alone (74 and 69, for people living with overweight and obesity, respectively ( Sach *et al.*, 2007)). The major impact on health-related quality of life is further highlighted by the health utility score, half that of the UK general population (0.48 vs 0.86) ( Ara & Brazier, 2011), and the high proportion of participants unable to carry out usual activities (10%), compared to 1% in the general population aged 35-64 in Europe including the UK ( Janssen *et al.*, 2021).

Most participants had not been vaccinated before becoming ill with COVID-19, with the number of affected organ systems (based on core symptoms and symptom group counts) lower by one in people infected after vaccination, in line with evidence linking vaccination with reduced LC severity ( Byambasuren *et al.*, 2023; Tran *et al.*, 2023).

Nearly all participants received healthcare support for LC through the NHS, mainly through GPs, Long COVID clinics, hospital specialist services and physiotherapy. A quarter of participants had turned to private healthcare, anecdotally because of difficulties accessing support through the NHS. Our qualitative analysis will further explore the reasons for accessing private healthcare and the additional burden on participants and their families.

Non-healthcare-related support was sought by every 3 of 5 participants, mostly through online LC communities or local support groups. Online LC communities evolved early in the pandemic, with patients sharing experiences of the after-effects of COVID-19 on social media and collectively coining the term "Long Covid" ( Callard & Perego, 2021). At a time of social distancing and with evidence about LC first emerging through these patient groups, online communities were an important source of support for LC patients, providing a safe space to speak about symptoms and concerns, filling a gap in care, and helping patients find validation in others sharing similar experiences ( Day, 2022; Russell *et al.*, 2022).

In the ReDIRECT study, the number of pre-existing comorbidities and the prevalence of overweight and obesity before LC corresponded to the average in the UK population ( Office for National Statistics UK Health Indicators, 2022; Organisation for Economic Co-operation and Development). Pre-existing asthma, hypertension and depression have been associated with increased risk for LC ( Loosen *et al.*, 2022; Sudre *et al.*, 2021; Tsampasian *et al.*, 2023), which may explain that the proportion of people with asthma in our study was greater than the age-standardised rate in the UK (18% vs 10% for men and 12% for women). While pre-COVID-19 weight data was available for only 79% of our participants, at least 71% were living with overweight or obesity prior to becoming ill with COVID-19. Interestingly, the proportion of pre-existing conditions generally associated with overweight and obesity, such as hypertension, dyslipidaemia, and type 2 diabetes, was about 5% (diabetes) to 10% (hypertension and dyslipidaemia) lower compared to the general population ( Office for National Statistics UK Health Indicators, 2022). Whether this is due to the lower mean age (46), the largely female population or other factors is unclear.

Previous studies have highlighted the impacts of the COVID-19 pandemic on health behaviours that influence weight (including eating habits, physical activity, stress, and sleep), with most reporting weight gain across populations ( Alah *et al.*, 2021; Almandoz *et al.*, 2022; Flanagan *et al.*, 2021; Khubchandani *et al.*, 2020; Khubchandani *et al.*, 2022; Mason *et al.*, 2022). The ReDIRECT study provides novel insight into body composition and weight management in this participant group living with both LC and overweight. Whilst a significant proportion of participants already lived with overweight or obesity prior to contracting COVID-19 (over two-thirds), a majority (68%) reported having gained a substantial amount of weight (5kg or more) following infection. The reasons for this weight gain may be multiple, including behavioural changes during confinement periods, compounded by fatigue and mobility issues, both common symptoms reported in this study. There is currently limited evidence exploring this in LC, and this aspect will be further explored in the ReDIRECT trial qualitative analysis.

Most participants were already familiar with weight management before enrolling in ReDIRECT, with 4 of 5 participants having previously tried losing weight and 3 of 5 having successfully lost weight at some point in their lifetime. Commercial weight loss programmes were the most popular and effective for losing weight, followed by increased physical activity and self-directed diet regimes. BMI was higher pre-COVID and at baseline for those who had previously attempted weight loss than those who had not. However, the weight gained post-COVID was not different between the two groups.

Behaviour changes were common after contracting COVID-19 infection. Notably, physical activity levels reduced from exercising occasionally (< 3 times per week) to walking only, with nearly a quarter of participants not exercising at all, at baseline. Attempts at dietary modifications were common, with over half modifying their diet after developing LC, commonly using approaches including calorie restriction and low carbohydrate diets as well as other diet modifications including anti-inflammatory, vegetarian/vegan, dairy-free, gluten-free, low sugar, low FODMAP, and low histamine diets, despite the lack of evidence base in this space. Similarly, dietary supplementation was common and varied. Changes and supplementation were often self-led, short-term, and without professional support. By baseline, most participants had returned to an omnivorous diet.

One of the proposed mechanisms underpinning LC is immune dysregulation and long-term systemic inflammation with elevated levels of inflammatory markers, such as interleukin (IL)-1β, IL-6, and TNF-α ( Peluso *et al.*, 2021; Schultheiss *et al.*, 2022; Schultheiss *et al.*, 2023; Zhang *et al.*, 2023). Anti-inflammatory therapies in LC patients are potential treatment strategies currently being studied in several clinical trials ( Bonilla *et al.*, 2023). Weight loss has been shown to reduce pro-inflammatory markers present in the chronic low-grade inflammatory state associated with obesity, including CRP, IL-6 and TNF-a ( Christiansen *et al.*, 2010; Marfella *et al.*, 2004; Moeller *et al.*, 2016) and may thereby help manage LC symptoms. Weight loss may also benefit people living with both LC and overweight/obesity beyond the effect on inflammation. The symptoms commonly reported in LC, including fatigue, shortness of breath, pain, and mood disorders, are also experienced by people living with obesity. Weight management trials conducted in study populations without LC observed that weight loss improved breathlessness ( Bernhardt & Babb, 2014; Stenius-Aarniala *et al.*, 2000), relieved joint pain ( Messier *et al.*, 2018; Riddle & Stratford, 2013), had a positive impact on cardiometabolic health and improved quality of life ( Avenell *et al.*, 2004).

### Strengths and limitations

A key strength of the ReDIRECT study is its design, which relies on remote data collection and intervention delivery. This is particularly important in the context of the population studied since people living with LC face a high fatigue and mobility burden, sometimes compounded by careful avoidance of higher-risk situations where COVID-19 exposure may be likely. Remote delivery allowed for a flexible approach to recruitment across the UK, increased sample diversity and potentially facilitated greater inclusion by removing the burden on participants linked to attending study centres or clinics to take part. The intervention was a repurposing of the evidence-based weight loss intervention used in the DiRECT study ( Lean *et al.*, 2018), known to be safe and effective in people with Type 2 diabetes and overweight/obesity. Using a pragmatic trial design, with professional support, also recognises that dietary choices and adherence are complex and need to be evaluated individually in real-life settings.

The ReDIRECT study relied, from its conception through to its execution, on in-depth co-production with patient representatives. This co-production is fully reflected in the breadth and depth of tools to support data capture and the trial's design to reflect the requirement for personalisation and adjusting how the intervention was delivered. As such, a unique feature of the study is the selection of a personalised primary outcome, whereby participants nominate the LC symptom they would most like to improve. To our knowledge, no other trial has used such an approach.

There is currently no proven treatment or rehabilitation for LC and limited research on dietary interventions. To our knowledge, this is the first study to investigate a dietary management approach to improve LC symptoms.

The study also has limitations. While the remote nature of data collection eased the burden of not attending study visits in person, it also increased the need for computer literacy and the burden linked to engaging with data capture forms for participants, linked to anecdotal study fatigue. ReDIRECT relies on self-measurement of weight and blood pressure and self-reports. Flexibility was built into questionnaire completion timelines to minimise missing data and alleviate participant burden with assistance via email, text messages and phone calls if required. Questionnaires were also carefully checked for missing answers, and entry mistakes were followed up with participants.

In total, five participants in our study reported their first COVID-19 infection before or around the time of the first COVID-19 confirmed case in the UK (January 26, 2020) ( The-nCoV Outbreak Joint Field Epidemiology Investigation Team & Li, 2020) in October and November 2019, and in January 2020. At that time, there was no testing available to confirm these infections. In all five cases, however, participants reported awareness of LC symptoms within 7 to 14 months of infection with LC diagnosed by healthcare professionals and also had a second (confirmed) infection in 2020-2022 before joining the study.

Despite focused efforts to reach out to a broad segment of the population, men are under-represented in this study, as are people from more socioeconomically disadvantaged backgrounds and minority ethnic groups. Recruitment through general practices was challenging, possibly linked to the limited use of clinical codes and challenges in identifying patients with LC ( Jeffrey *et al.*, 2023; Walker *et al.*, 2021). Several factors may also have contributed to our study's high proportion of women. Being a woman is associated with 50% higher odds of developing LC ( Su *et al.*, 2022; Subramanian *et al.*, 2022; Thompson *et al.*, 2022; Tsampasian *et al.*, 2023; Whitaker *et al.*, 2022). The PHOSP study also found that women were less likely to have recovered from COVID-19 after one year (OR 0.68) ( Evans *et al.*, 2022). Finally, past studies have shown that structured weight loss programmes, such as the Counterweight-Plus programme, are largely used by women ( Robertson *et al.*, 2014; Stubbs *et al.*, 2015; Tudor *et al.*, 2021).

## Conclusions

The baseline characteristics of the ReDIRECT study participants outline a complex presentation of symptoms attributable to LC but also to having excess weight, which increased substantially during the pandemic. With excess weight being a risk factor for LC and a large proportion of the participants having attempted dietary changes, often without support, in the context of their infection and subsequent LC, there is an urgent need to develop evidence supporting dietary strategies for people living with both LC and overweight, recognising that such approach will need a high level of personalisation to meet the needs of the individuals.

## Data Availability

As per our study protocol, access to the raw data is restricted to the primary research team whilst the research is being conducted (until the end of the project, May 2024) and publication of the primary research papers. Upon publication of these papers, fully anonymised data will be placed on a research data repository with access given to researchers on request to the corresponding authors and subject to appropriate data sharing agreements. Figshare: ReDIRECT study eCRF questionnaires screenshots. https://doi.org/10.6084/m9.figshare.21270837 ( Combet *et al.*, 2022). Figshare: ReDIRECT Baseline Summary Tables and Figures. https://doi.org/10.6084/m9.figshare.24602367 ( Haag *et al.*, 2023b). Data are available under the terms of the Creative Commons Zero "No rights reserved" data waiver (CC0 1.0 Public domain dedication).
