# Supplementary material for: Baseline Characteristics in the Remote Diet Intervention to REduce long-COVID Symptoms Trial (ReDIRECT)
Source: NIHR Open Res. 2024 Mar 5;4:7. [Version 1] doi: 10.3310/nihropenres.13522.1 (PMC11320183; doi:10.3310/nihropenres.13522.1)
Supplement: Peer review history

# Reviewer report: Reviewer response for version 1

**DOI:** 10.3310/nihropenres.14676.r32176

**Published:** 2024-08-19

**Authors:** Yessica Abigail Tronco Hernández

**Affiliations:** 1 University of Plymouth, Plymouth, England, UK

This is a good article that attempts to cover comprehensively the health profile of people with long Covid to deliver a dietary intervention. Some suggestions to improve follow:

 ABSTRACT

 It is worth mentioning in the methods section that the work was conducted in the UK or only in England and Scotland

 Mention what kind of data analysis was conducted

 It is not clear whether they gained weight which led to obesity or if they had obesity before they had their first covid infection

 From the abstract it is not clear whether this is a protocol or the trial finalised. This should be clarified to avoid misleading the reader. The title says baseline characteristics but this is not clear in the abstract

 The lay summary suggests it is a protocol, but the study seems to be a trial when looking at some results.

 If an acronym is used for long covid, which is the main interest variable, then why not using one for covid-19?

 Introduction

 End of paragraph 2, define what you mean by long term

 6 ^th^ paragraph, Is Bakaloudi reporting UK populations? And how do you discard those who had obesity pre pandemic to present figures? Need to clarify

 Methods

 Please provide a reference for the GRIPP2

 It is not clear whether participants received any compensation for their time

 Need to clarify what is your rationale for selecting the tools for XYZ symptoms, on the other hand to clarify whether these tools were selected after participants answered the baseline questionnaire and selected the most burdensome symptoms. The first paragraph of page 8 does not explicitly say this.

 RESULTS

 Need to specify for those decreased work hours whether this was due to their own disease or due to lockdown conditions or other reasons, to avoid cofounders or speculation

                

 DISCUSSION, limitations and conclusions

 This is the section I enjoyed the most as you reviewed several papers. More data on the influence of the pandemic on health and wellbeing during lockdowns (Tronco Hernandez et al, 2021) as well as experiences reported by people with long COVID and lessons for professionals and researchers (Tronco Hernandez et al. 2023) would provide more breadth to this body of evidence

 Good to acknowledge the major limitation self reports and lack of training to self-measure weight and blood pressure

 The burden of the questionnaire on participants needs to be considered too

 In general, researchers studied a lot of variables, some may be cofounders and lead to spurious assumptions. I suggest you divide them into primary (weight, blood pressure, etc) and secondary variables (e.g. socioeconomic status, vaccination status) otherwise there is too much data hence the main aim can get easily lost for readers or they could make erroneous assumptions

Is the work clearly and accurately presented and does it cite the current literature?

Partly

If applicable, is the statistical analysis and its interpretation appropriate?

I cannot comment. A qualified statistician is required.

Are all the source data underlying the results available to ensure full reproducibility?

Partly

Is the study design appropriate and is the work technically sound?

Yes

Are the conclusions drawn adequately supported by the results?

Partly

Are sufficient details of methods and analysis provided to allow replication by others?

Partly

Reviewer Expertise:

I am a postdoctoral researcher who has published several papers on the effects of covid on people and how to best support professionals and employers delivering wellbeing and dietary strategies to patients with Long covid

I confirm that I have read this submission and believe that I have an appropriate level of expertise to confirm that it is of an acceptable scientific standard, however I have significant reservations, as outlined above.

## References

[1] : Influence of the COVID-19 Lockdown on the Physical and Psychosocial Well-being and Work Productivity of Remote Workers: Cross-sectional Correlational Study. *JMIRx Med* .2021;2(4) : 10.2196/30708 e30708 10.2196/30708 34898665 PMC8641476

[2] : Providing optimal nutritional care to patients with long-COVID. *Primary Health Care* .2023;33(4) : 10.7748/phc.2023.e1785 30-35 10.7748/phc.2023.e1785

# Reviewer report: Reviewer response for version 1

**DOI:** 10.3310/nihropenres.14676.r32175

**Published:** 2024-07-08

**Authors:** Jessica Campbell

**Affiliations:** 1 Auckland University of Technology, Auckland, New Zealand

Agreeing with peer reviewer 1, this is a well written and interesting paper which I enjoyed reading. It would however benefit from some clarification regarding the aim of the paper. In particular, when reading the abstract, it was not initially clear that results would not be presented, though the title of the paper is clear. Additionally, below are a few comments/suggestions for the authors to consider.

1. Add details regarding which version of R was used.
2. Clarify the use of statistical tests. For example, were Mann-Whitney tests chosen due to data failing the assumptions of parametric testing, or was this always intended to be the test of choice?
3. Throughout the paper it would be helpful to make a clearer distinction between acute Covid-19 infection and Long Covid. If the authors have data, it would be interesting to see more detailed information about when LC started (rather than just how long individuals had been experiencing LC symptoms). Data are presented relating to the dates of each acute Covid-19 infection and about these infections in relation to vaccination; however, it would have been nice to see these events in relation to LC. For example, was LC more likely after the first or second infection? Was it more likely before or after vaccination? This wasn’t entirely clear.
4. Similar to point 3, there is discussion throughout the paper about changes after Covid-19 (for example, weight gain). It would be good to clarify whether this relates to LC specifically, or to the first acute infection, the second acute infection, etc. In some cases, this may be difficult to determine, but acknowledgment of this would be beneficial.
5. I suggest changing ‘vegan’ to ‘plant-based’ given that the former is an ethical stance related to broader changes in lifestyle, whereas the latter refers specifically to a diet which may be adopted for health reasons (as is more appropriate in this context).
6. It is perhaps worth noting or clarifying that some of the changes that occurred post-Covid may be related to more general pandemic responses rather than being (only) related to illness. For example, weight gain and reductions in exercise were widely reported due to lockdown measures even in otherwise healthy individuals. If it is possible to rule this out, a comment noting this would be beneficial.
7. While (online) LC support groups were undoubtedly helpful for many, it may be worth noting the downsides of these groups e.g. (Turner J., 2017)
8. While the limitations section notes that self-report measures were used, this could be explained more explicitly.
9. The discussion mentions that several patients who reported infection before testing was widely available became aware of LC within 7 to 14 months post-infection. This point may need further elaboration. Rather than focusing on the lack of testing, consider discussing the significant time gap between infection and the onset of LC symptoms. This delay makes it challenging to ascertain whether the symptoms are genuinely related to Covid-19 or if they might be idiopathic.

Is the work clearly and accurately presented and does it cite the current literature?

Yes

If applicable, is the statistical analysis and its interpretation appropriate?

I cannot comment. A qualified statistician is required.

Are all the source data underlying the results available to ensure full reproducibility?

Yes

Is the study design appropriate and is the work technically sound?

Yes

Are the conclusions drawn adequately supported by the results?

Yes

Are sufficient details of methods and analysis provided to allow replication by others?

Yes

Reviewer Expertise:

I have previously published research on Covid-19 and currently focus on studying the effects of nutritional interventions. My work involves exploring how diet and lifestyle changes can impact health outcomes, particularly in the context of chronic disease.

I confirm that I have read this submission and believe that I have an appropriate level of expertise to confirm that it is of an acceptable scientific standard.

## References

[1] : Online Support Groups: The Good, the Bad, and the Motivated. *Journal of Consumer Health on the Internet* .2017;21(1) : 10.1080/15398285.2017.1279930 11-25 10.1080/15398285.2017.1279930

# Reviewer report: Reviewer response for version 1

**DOI:** 10.3310/nihropenres.14676.r32173

**Published:** 2024-07-04

**Authors:** Teresa Maria Tomasa-Irriguible

**Affiliations:** 1 Hospital Universitari Germans Trias i Pujol, Badalona, Spain

**Reviewer Comments. Baseline Characteristics in the Remote Diet Intervention to REduce long-COVID Symptoms Trial (ReDIRECT)**

**ABSTRACT**

- Methods section:
  - Does not reflect what the paper is about. What is the objective/s? “Baseline Characteristics”? You could write this sentence: “Here, we describe the baseline characteristics of ReDIRECT participants, focusing on socioeconomic and demographic factors as well as dietary and medical history, including LC diagnosis and symptoms”.
- Conclusions.
  - Does not answer the title “ **Baseline Characteristics”** as no objectives are described at M&M…

**MAIN SECTIONS**

**INTRODUCTION**

- **4 ^th^ paragraph, 1 ^st^ sentence. **Reference missing. This point is important as your hypothesis states that obesity is a risk factor for LC.
- **5 ^th^ paragraph, 1 ^st^ sentence **Reference missing. This point is important, as your hypothesis is whether a dietary modification may influence LC.
- **Last paragraph. **This is the real M&M. This text should also be at the abstract at introduction and M&M sections.

**METHODS**

- **2 ^nd^ paragraph, 1 ^st^ sentence. **I think that could be a problem, since they may not offer a fairness judgment.
- ***Ethical approval*** **.** **3 ^rd^ paragraph. **Think about moving this paragraph to 3 paragraphs below, in ***Study design and participants*** before "A total of 240 participants..."
- ***Study design and participants*** ***.*** **Last paragraph, last sentence.** One question: “Further support is provided through in-app weekly monitoring and nudges, personalized messaging, and group support” this was also done with the control group in the first 3 months and until de year?

**RESULTS**

- **Long COVID**
  - **1 ^st^ paragraph, 2 ^nd^ sentence. **This is a very important epidemiological finding! Hence, think about writing this finding in the abstract and conclusions sections.
  - **7 ^th^ paragraph, 2 ^nd^ sentence. Regarding cognitive impairment. **This is also a relevant epidemiological finding; Moreover, this symptom does not depend a priori on the patient's weight and other confusing symptoms. Think about writing this finding in the abstract and conclusions sections.
  - **8 ^th^ paragraph, 2 ^nd^ sentence.** This is also a very important epidemiological finding. Think about writing this finding in the abstract and conclusions sections.
  - **Weight management and diet.** **1 ^st^ and 2 ^nd^ paragraphs. **Some of these findings are relevant and should be in the abstract and conclusion section. In addition, as you mentioned along the manuscript, median weight gain of 11.5 kg by itself can cause fatigue, dyspnea and depression. Hence, you would argue this thing in the discussion section.
  - **Weight management and diet.** **6 ^th^ paragraph. **These findings are relevant and should be in the abstract and conclusion section.

**DISCUSSION**

  

- **2 ^nd^ paragraph. **This paragraph is already in the Results section. No comparison is done with other studies. This paragraph could be removed from here.

 

- **6 ^th^ paragraph.** This is also in the Results section. No comparison is made with other studies. This paragraph could be removed from here, too.

 

- **7 ^th^ paragraph. **This is also in the Results section. No comparison is made with other studies. This paragraph could be removed from here, too.

 

- **One question for the sentence at the end of 8 ^th^ paragraph. **What was the average age in the Office for National Statistics UK Health Indicators, 2022?

 

- **10 ^th^ paragraph. **This is also in the Results section. No comparison is made with other studies. This paragraph could be removed from here, too.

 

- **11 ^th^ paragraph. **This is also in the Results section. No comparison is made with other studies. This paragraph could be removed from here, too.

 

- Instead of this paragraphs, you could discuss something about this other issues in **Long COVID **results:
  - **1 ^st^ paragraph, 2 ^nd^ sentence. **This is a very important epidemiological finding! Hence, think about writing this finding in the abstract and conclusions sections.
  - **7 ^th^ paragraph, 2 ^nd^ sentence. Regarding cognitive impairment. **This is also a relevant epidemiological finding; Moreover, this symptom does not depend a priori on the patient's weight and other confusing symptoms. Think about writing this finding in the abstract and conclusions sections.
  - **8 ^th^ paragraph, 2 ^nd^ sentence.** This is also a very important epidemiological finding. Think about writing this finding in the abstract and conclusions sections.
  - **Weight management and diet.** **1 ^st^ and 2 ^nd^ paragraphs. **Some of these findings are relevant and should be in the abstract and conclusion section. In addition, as you mentioned along the manuscript, median weight gain of 11.5 kg by itself can cause fatigue, dyspnea and depression. Hence, you would argue this thing in the discussion section.
  - **Weight management and diet.** **6 ^th^ paragraph. **These findings are relevant and should be in the abstract and conclusion section.

**CONCLUSIONS**

**2 ^nd^ sentence. **You did exclude the thin patients by design of the study. This sentence cannot be a conclusion of this **Baseline Characteristics** epidemiological study.

 Instead of this sentence, you could conclude something more concrete about this **Long COVID **results:

-
  - **1 ^st^ paragraph, 2 ^nd^ sentence. **This is a very important epidemiological finding! Hence, think about writing this finding in the abstract and conclusions sections.
  - **7 ^th^ paragraph, 2 ^nd^ sentence. Regarding cognitive impairment. **This is also a relevant epidemiological finding; Moreover, this symptom does not depend a priori on the patient's weight and other confusing symptoms. Think about writing this finding in the abstract and conclusions sections.
  - **8 ^th^ paragraph, 2 ^nd^ sentence.** This is also a very important epidemiological finding. Think about writing this finding in the abstract and conclusions sections.
  - **Weight management and diet.** **1 ^st^ and 2 ^nd^ paragraphs. **Some of these findings are relevant and should be in the abstract and conclusion section. In addition, as you mentioned along the manuscript, median weight gain of 11.5 kg by itself can cause fatigue, dyspnea and depression. Hence, you would argue this thing in the discussion section.
  - **Weight management and diet.** **6 ^th^ paragraph. **These findings are relevant and should be in the abstract and conclusion section.

Is the work clearly and accurately presented and does it cite the current literature?

No

If applicable, is the statistical analysis and its interpretation appropriate?

Not applicable

Are all the source data underlying the results available to ensure full reproducibility?

Partly

Is the study design appropriate and is the work technically sound?

No

Are the conclusions drawn adequately supported by the results?

No

Are sufficient details of methods and analysis provided to allow replication by others?

Partly

Reviewer Expertise:

ORCID https://orcid.org/0000-0001-8033-8527

I confirm that I have read this submission and believe that I have an appropriate level of expertise to confirm that it is of an acceptable scientific standard, however I have significant reservations, as outlined above.

# Reviewer report: Reviewer response for version 1

**DOI:** 10.3310/nihropenres.14676.r31535

**Published:** 2024-05-31

**Authors:** Hiyam Al-Jabr

**Affiliations:** 1 University of Keele, Keele, UK

This is a well-written exciting paper, thank you for writing this.

 I have few comments for authors to consider:

 1. The aim is not clear in the abstract. Please add the aim of the study to the abstract.

 2. Please clarify aim on page 4, I was confused whether the paper is going to talk about the impact of the intervention on listed outcomes or just describe characteristics of the study population, please clarify the exact aim of the paper.

 3. Please define abbreviations of "UoG" and "PPI" on page 4.

 4. On page 5, you could remove the section that starts with "The REC favorable opinion...." to "except where explicitly specified otherwise". this is by default a requirement by ethics whenever an NHS site is included.

 5. Could you please whether the answers to the "living situation" in table 2 is "select all that applies" as total number for this part exceeds 234 participants.

 6. on page 16, the sentence "To our knowledge, this is the first study to investigate a dietary management approach to improve LC symptoms". the paper only reports baseline characteristics of included participants, thus I'm not sure this line fits with this paper, especially that no impacts are mentioned related to the use of the intervention.

Is the work clearly and accurately presented and does it cite the current literature?

Yes

If applicable, is the statistical analysis and its interpretation appropriate?

I cannot comment. A qualified statistician is required.

Are all the source data underlying the results available to ensure full reproducibility?

Yes

Is the study design appropriate and is the work technically sound?

Yes

Are the conclusions drawn adequately supported by the results?

Yes

Are sufficient details of methods and analysis provided to allow replication by others?

Yes

Reviewer Expertise:

I'm a research Fellow with experience of conducting research in Long COVID.

I confirm that I have read this submission and believe that I have an appropriate level of expertise to confirm that it is of an acceptable scientific standard.
